# Care Pathways for Metabolic Dysfunction‐Associated Steatotic Liver Disease (MASLD): A State‐of‐The‐Art Review

**DOI:** 10.1111/liv.70603

**Published:** 2026-03-19

**Authors:** Kirsi M. A. van Eekhout, Leonard D. Broekman, Vivian D. de Jong, Maurice Michel, Rick Grobbee, Douglas Maya‐Miles, Manuel Romero‐Gómez, Jean Muris, Juan M. Mendive, Yasaman Vali, Oscar H. Franco, Jörn M. Schattenberg, Céline Fournier‐Poizat, Manuel Castro Cabezas, Maarten E. Tushuizen, Adriaan G. Holleboom, the GRIPonMASH consortium

**Affiliations:** ^1^ Department of Vascular and Internal Medicine Amsterdam University Medical Centre, University of Amsterdam Amsterdam the Netherlands; ^2^ Department of Global Public Health & Bioethics, Julius Center for Health Sciences and Primary Care University Medical Center Utrecht Utrecht the Netherlands; ^3^ Julius Clinical Zeist the Netherlands; ^4^ Department of Internal Medicine II Saarland University Medical Center, Saarland University Homburg Germany; ^5^ Saarland University Saarbrücken Germany; ^6^ SeLiver Group, Instituto de Biomedicina de Sevilla/CSIC/Virgen del Rocío University Hospital University of Seville, Seville, Spain/Centro De Investigación Biomédica en Red de Enfermedades Hepáticas y Digestivas (CIBEREHD) Seville Spain; ^7^ UCM Digestive Diseases. Virgen del Rocio University Hospital, Institute of Biomedicine of Seville, CIBEREHD University of Seville Seville Spain; ^8^ Care and Public Health Research Institute (CAPHRI), Department of Family Medicine Maastricht University Maastricht the Netherlands; ^9^ Department of Family Medicine, La Mina Primary Health Care Academic Centre University of Barcelona Barcelona Spain; ^10^ Department of Epidemiology and Data Science Amsterdam University Medical Centers Amsterdam the Netherlands; ^11^ Echosens Paris France; ^12^ Department of Internal Medicine Franciscus Gasthuis & Vlietland Rotterdam the Netherlands; ^13^ Department of Internal Medicine, Erasmus MC Rotterdam the Netherlands; ^14^ Department of Gastroenterology and Hepatology Leiden University Medical Centre, University of Leiden Leiden the Netherlands

**Keywords:** care pathway, chronic liver disease, liver fibrosis, metabolic dysfunction‐associated steatohepatitis, metabolic dysfunction‐associated steatotic liver disease

## Abstract

Metabolic dysfunction‐Associated Steatotic Liver Disease (MASLD) is a growing clinical challenge, necessitating effective diagnostic strategies to identify advanced liver fibrosis while minimising unnecessary referrals of mild cases. Current clinical guidelines recommend care pathways utilising non‐invasive tests (NITs) to stratify patients, but the optimal diagnostic algorithm across care settings remains unclear. This state‐of‐the‐art review systematically examines studies describing clinical care pathways for detecting advanced fibrotic MASLD and stratifying patients at risk. A comprehensive literature search of MEDLINE, Embase, Cochrane Library, and Scopus, finalised in January 2026, identified nine relevant studies that met predefined criteria including structured care plans and applicability beyond diagnosis alone. Pathway populations included patients at risk for MASLD (type 2 diabetes (*n* = 4) or broad range cardiometabolic risk factors (*n* = 1)) or confirmed MASLD (*n* = 4). The most frequently employed NITs were FIB‐4 and vibration‐controlled transient elastography (VCTE). Numbers needed to screen (NNS) for hepatology referral and advanced fibrosis detection varied considerably across pathways and populations, reflecting heterogeneity in design and fibrosis assessment methods. All studies reported improved patient risk stratification; attendance rates declined at each pathway step. Findings suggest that NIT‐based clinical care pathways can effectively align patient management and optimise transmural care for MASLD. Nonetheless, heterogeneity in pathway design and fibrosis determination highlights the need for standardised protocols and validation in larger, at‐risk cohorts to strengthen evidence supporting widespread adoption. This review contributes to advancing MASLD management within evolving clinical frameworks.

## Key Points

- NIT‐based care pathways improve identification of advanced MASLD and reduce unnecessary hepatology referrals.
- Considerable heterogeneity exists in pathway design, primarily in terms of target populations, NITs, and definitions of advanced disease, resulting in a widely varied number needed to screen.
- Present literature underscores the need for a universally accepted outcome framework for MASLD care pathways.
- Large‐scale, standardised, real‐world studies are urgently needed to guide scalable implementation.

**Abbreviations**
AASLD: American Association for the Study of Liver Disease
AGA: American Gastroenterological Association
ALD: Alcohol‐related liver disease
APRI: Aspartate transferase to platelet ratio index
ELF: Enhanced liver fibrosis
FIB‐4: Fibrosis‐4
GBD: Global burden of disease
HCC: Hepatocellular carcinoma
LSM: Liver stiffness measurement
MASH: Metabolic dysfunction‐associated steatohepatitis
MASLD: Metabolic dysfunction‐associated steatotic liver disease
MetALD: Metabolic dysfunction‐associated and alcohol‐related liver disease
NFS: Non‐alcoholic fatty liver disease fibrosis score
NITs: Non‐invasive tests
NNS: Number needed to screen
OR: Odds ratio
SWE: Shear wave elastography
T2DM: Type 2 diabetes mellitus
VCTE: Vibration‐controlled transient elastography

## 1 Introduction

The prevalence of Metabolic dysfunction‐Associated Steatotic Liver Disease (MASLD) is rising. The global prevalence of MASLD has increased from 25.3% to 38.2% over the past three decades and is currently the most common cause of chronic liver disease [1, 2]. In patients with type 2 diabetes mellitus (T2DM), the prevalence is as high as 60%–80% [3, 4]. MASLD is currently a leading cause of liver‐related morbidity and mortality and is independently associated with an increased risk of cardiovascular events [5].

In the majority of cases, MASLD remains in a mild, early, and stable stage, namely isolated hepatic steatosis. However, in about 12%–40% of cases, MASLD progresses to Metabolic dysfunction‐Associated Steatohepatitis (MASH), a more severe stage with liver inflammation. This progression often goes unnoticed due to a lack of symptoms in patients and a lack of awareness in both patients and care providers. As a result, MASH may further progress to MASH‐fibrosis (in 6.5%–19.1%), −cirrhosis (in 3%–5%), and hepatocellular carcinoma (HCC) [6]. The excess risk of all‐cause 20‐year mortality compared to controls from the general population increases rapidly with the progression of MASLD, climbing from 10.7% to 25.6% for fibrotic‐MASH and to 49.4% for cirrhosis [7].

The rising prevalence of MASLD and MASH poses a major burden on healthcare systems and society globally [4]. Recent data from the Global Burden of Disease (GBD) study on mortality and disability in several countries indicate a rapidly increasing health and economic burden of MASLD in many parts of the world [2, 8].

This growing impact of disease associated with MASLD‐MASH demands a clear‐cut diagnostic approach. However, in current practice there is still a significant variability, leading to both over‐ and underdiagnosis. In combination with highly prevalent cardiometabolic comorbidities, this highlights the need for integrated, multidisciplinary, and holistic care throughout all levels of care [9]. In that light, the implementation of a MASLD care pathway may facilitate improved case finding of patients with advanced fibrotic MASLD, while simultaneously reducing unnecessary hepatology referrals of mild stages. This demand is intensifying with the recent FDA‐approval of Resmetirom as a treatment for non‐cirrhotic MASH with moderate to advanced liver fibrosis, as well as the expected approval of other novel therapies like incretin‐based drugs in the near future [4, 10, 11].

Various non‐invasive tests (NITs) are being developed and validated to detect advanced fibrosis, reducing the need for invasive liver biopsy of which a selection is already being widely used in clinical practice [12, 13]. However, no single NIT has yet been able to fully replace liver biopsy. To improve detection and risk stratification, NITs are being combined into one‐, two‐ or three‐tiered pathways. Such sequenced algorithms are increasingly being recommended in both national and international guidelines. Indeed, the recently updated (June 2024) EASL‐EASD‐EASO clinical practice guideline on the management of MASLD advocates a two‐tiered pathway for risk assessment, beginning with the Fibrosis‐4 (FIB‐4) score, followed by a Vibration‐Controlled Transient Elastography (VCTE/FibroScan) liver stiffness measurement (LSM) [14].

However, the optimal method for early detection of advanced fibrotic MASLD, as well as the most appropriate population to screen, remains unclear and continues to be a subject of debate. There is also ongoing debate about the optimal thresholds for referral and further testing, leading to inconsistencies in real‐world implementation. The aim of this state‐of‐the‐art review is therefore to provide a comprehensive and clear overview of published data on clinical care pathways aimed at detection and risk stratification of fibrosis in patients with, or at risk for, MASLD.

## 2 Methods

To maintain uniformity, the new nomenclature terms MASLD and MASH will be applied throughout this review. This also applies to descriptions of studies published under the previous nomenclature of NAFLD and NASH [15, 16], as the criteria are identical for 99% of patients [15]. The full protocol for this review was published and registered with Open Science Framework (OSF) [17].

### 2.1 Inclusion/Exclusion Criteria

For an article to be eligible for inclusion, the study had to describe one or more clinical care pathways aimed at screening for significant (≥ F2) or advanced (≥ F3) fibrosis in adult patients (≥ 18 years). As such, studies including patients with confirmed MASLD as well as those at risk for MASLD (e.g., patients with metabolic syndrome) were deemed eligible. Articles were eligible if they described a clinical care pathway that met the following criteria: a well‐defined patient population, a well‐defined, structured plan of care, and applicability to multiple aspects of care (and not merely diagnosis). The care pathway setting could be in primary care, an outpatient clinic, or a combination of both.

Articles focusing on strategies for detecting other liver diseases (e.g., viral, alcohol‐related) or evaluating a strategy for detection of advanced liver disease in general (e.g., grouping together alcohol‐related liver disease (ALD), metabolic dysfunction‐associated and alcohol‐related liver disease (MetALD), and MASLD) were excluded, as well as prevalence studies and retrospective studies. To provide a comprehensive overview of all available articles, no time or language filter was applied. Only articles with full text availability were included, for example, abstracts or poster presentations were excluded. When multiple publications were published in relation to the same care pathway, the most comprehensive paper providing a complete description of the pathway was selected.

### 2.2 Search Strategy

The search strategy was developed in collaboration with research librarians at the University of Amsterdam. For the full Ovid MEDLINE search see Supplementary S1. The search terms were deliberately broad so as not to miss any relevant studies, including those published under the previous nomenclature. The search strategy was applied in the following databases: MEDLINE, Embase, Cochrane Library, and Scopus. The database screening was finalised on January 19th, 2026. Additionally, a manual search of clinical guidelines and reference lists of eligible publications was conducted to further extend the search.

### 2.3 Study Selection

After finalising the search strategy in all databases, the records identified were combined and duplicates were removed. For the screening and selecting of the studies, Rayyan software was utilised [18]. Data were initially screened by title and abstract by two reviewers (KvE and LB) independently and with blinding to the others' decisions in place. After initial screening, blinding was removed and any conflicting decisions were discussed, after which the reviewers proceeded to full text screening. Again, performed independently and under blinded conditions. After full text screening, any conflicting decisions were debated. If disagreements persisted, a third reviewer (AH) was consulted.

### 2.4 Data Extraction

Data were extracted from the selected articles into a standardised form including the following items: general study information (author, year of publication, country); pathway information (setting of recruitment, NITs used in pathway, subsequent steps, follow‐up plan); study population (included patients, number of patients, control group, number in control group); outcome details (primary and secondary outcomes). Further data extracted included the number needed to screen (NNS) for hepatology referral, used definitions for significant or advanced fibrosis, the NNS for detecting one case of significant or advanced fibrosis, and data regarding attendance rate, since these were seen as primary results of interest reflecting the clinical performance of a care pathway. The NNS for referral and the NNS for detection of a case of significant or advanced fibrosis were calculated based on the numbers presented in the article to ensure a certain degree of homogeneity in outcome measures and hence allow for more adequate comparison of care pathway performance.

## 3 Results

### 3.1 Literature Search

**FIGURE 1:**
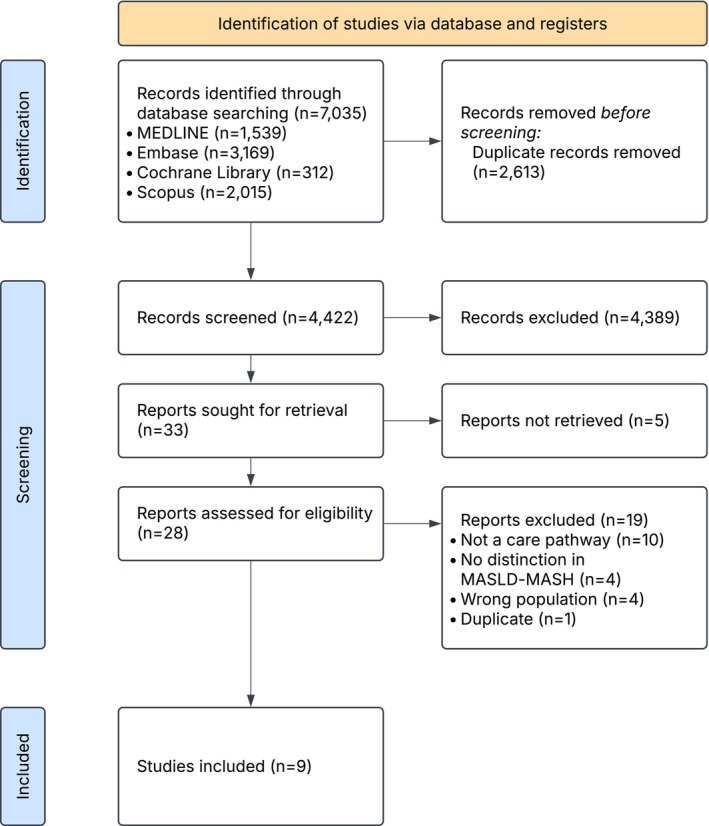
PRISMA flowchart of screening process.

A total of 7035 records were identified through database searching. The literature search and selection process for this review are illustrated in a PRISMA flowchart (Figure 1). After removing the duplicates, 4422 records remained and were screened by title and abstract. Thirty‐three records underwent full‐text screening for eligibility. Twenty‐four studies were excluded after full‐text review, mostly because they did not meet the predefined definition of a clinical care pathway. Overall, nine studies were eligible for inclusion [19, 20, 21, 22, 23, 24, 25, 26]. The included studies involved seven countries (three from the United States, two from the United Kingdom, one from Canada, one from Australia, one from France, and one from Hong Kong & Malaysia). All studies were published between 2019 and 2025.

### 3.2 Pathway Description

**FIGURE 2:**
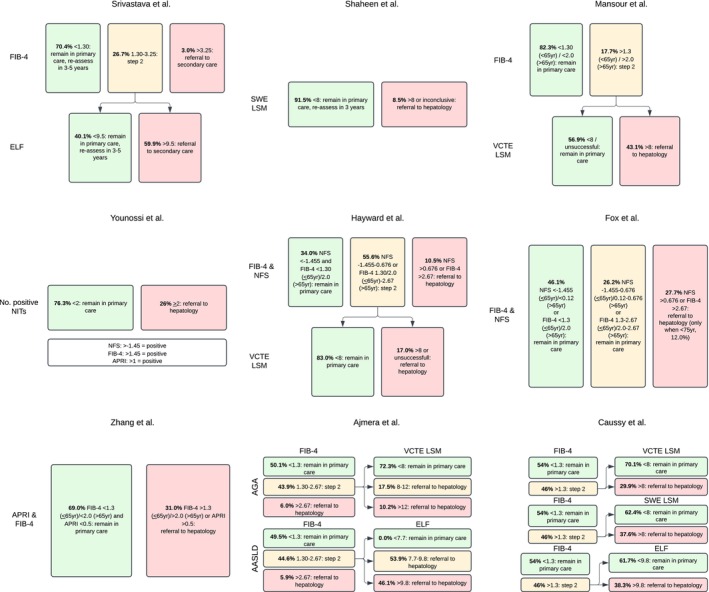
Visual representation of the high degree of variety in care pathway design. Studies are presented in order of year of publication (early to late). AASLD = American Association of the Study of Liver Disease; AGA = American Gastroenterological Association; APRI = aspartate transferase to platelet ratio index; ELF = enhanced liver fibrosis score; FIB‐4 = fibrosis‐4 score; LSM = liver stiffness measurement; NIT = non‐invasive test(s); NFS = non‐alcoholic fatty liver disease fibrosis score; SWE = shear wave elastography; VCTE = vibration‐controlled transient elastography.

**FIGURE 3:**
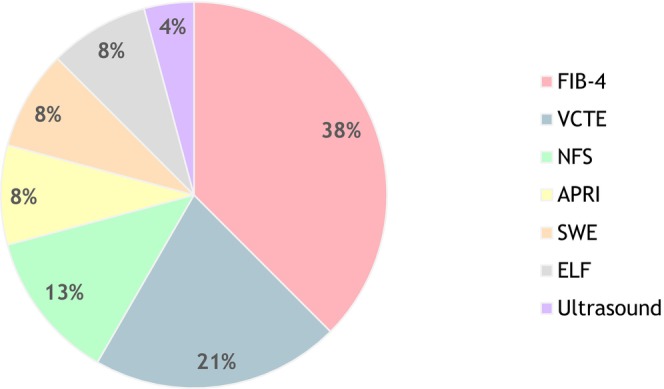
Distribution of NIT use across all studies (*n* = 24). APRI = aspartate transferase to platelet ratio index; ELF = enhanced liver fibrosis score; FIB‐4 = fibrosis‐4 score; NFS = non‐alcoholic fatty liver disease fibrosis score; SWE = shear wave elastography; VCTE = vibration‐controlled transient elastography.

Each article described a care pathway, including one or more NITs to identify patients with MASLD at risk for fibrosis. Table 1 and Figure 2 present an overview of the nine articles included in the review. Four studies used a one‐tier pathway, and five used a two‐tier pathway. FIB‐4 was the most often utilised NIT (see Figure 3), followed by VCTE‐measurement. Cut‐off values used in each study for each respective NIT varied and are presented in Figure 2. Five studies included patients at‐risk for MASLD, defined by the presence of a specific morbidity or risk factor: mainly T2DM (four studies) and one studie a broader range of cardiometabolic risk factors. Four studies included patients with confirmed MASLD based on steatosis detected by ultrasound. The study sizes varied from 162 included patients to a size of 2206 patients, with a total of 8551 participants across all studies. Eight of the nine studies were conducted in a primary care setting, with three of them also including outpatient care settings, and one study was exclusively conducted in an outpatient care setting. Three studies used a control group, which in every case consisted of patients receiving regular care.

**TABLE 1:** Study characteristics. Studies are presented in order of year of publication (early‐late).

Authors, year, country	Study size	Inclusion criteria and setting	Referral to hepatologysee Figure 2	NNS for hepatology referral	Definition significant or advanced fibrosis^a^	NNS to detect fibrosis	Control group	Service uptake	Further findings
Srivastava et al. [24] UK	*n* = 1452	Established MASLD diagnosis, based on Read code in primary care	FIB‐4 > 1.3 and ELF > 9.5 or FIB‐4 > 3.25	6	Advanced fibrosis Defined by liver biopsy, FibroScan (cut‐off not declared), or radiological features of cirrhosis	33	Yes Patients referred prior to introduction of the pathway in participating practices and those referred using standard care from non‐participating practices	55.3% (152/275) of high‐risk patients were actually referred to a hepatologist	OR 4.9 for detection of advanced fibrosis and cirrhosis compared to standard care within participating practices, OR 5.2 compared to standard care in non‐participating practices 81% reduction in unnecessary referrals compared to standard care
Shaheen et al. [23] Canada	*n* = 1958	MASLD confirmed by steatosis on US in patients with ≥ 1 CRF in primary care	SWE‐LSM ≥ 8 kPa	12	N.A.	N.A.	No	Not reported	3.4% high risk and referred to hepatology, 5.1% inconclusive and referred
Mansour et al. [22] UK	*n* = 466	T2DM and > 35 years in primary care	FIB‐4 > 1.3 (< 65 year)/> 2.0 (≥ 65 year) and VCTE‐LSM ≥ 8 kPa	19	Advanced fibrosis Defined by radiological evidence of cirrhosis, oesophageal or gastric varices, F3/F4 on liver histology or diagnosis based on overall clinical assessment	25	Yes Patients referred prior to introduction of the pathway	7% referred for VCTE did not attend or declined the test. Attendance rate was higher when VCTE was available at own GP surgery than a hospital clinic (100% vs. 25%).	OR 6.7 for detection of advanced liver disease compared to standard care in participating clinics 45.5% of patients with advanced liver disease had a normal ALT
Younossi et al. [27] USA	*n* = 716	T2DM + hypertension/dyslipidemia/BMI ≥ 30 kg/m^2^/elevated AST or ALT/steatosis on imaging in primary care & outpatient clinic	≥ 2 positive NITs (NFS > −145/FIB‐4 > 1.45/APRI > 1)	4	Significant fibrosis Defined by VCTE LSM ≥ 8.0 kPa	40	No	56.0% who were deemed high risk for agreed to be referred to hepatology	7.8% of high‐risk patients had liver stiffness ≥ 12 kPa
Hayward et al. [21] Australia	*n* = 162	MASLD confirmed by steatosis on US and/or abnormal AST or ALT in the presence of ≥ 1 CRF in primary care	NFS −1.455‐0.676 or FIB‐4 > 1.3 (< 65 year)/> 2.0 (≥ 65 year) and VCTE‐LSM ≥ 8.0 kPa Or NFS > 0.676 or FIB‐4 > 2.67	5	Significant fibrosis Defined by VCTE LSM ≥ 8.0 kPa	12	No	1.0% of patients requiring VCTE declined & discharged to primary care, 7.9% of referred patients failed to attend/declined & discharged to primary care	Higher prevalence of clinically significant fibrosis in T2DM (15.9% versus 1.4%), 35.7% of advanced fibrosis scored low‐risk FIB‐4
Fox et al. [20] USA	*n* = 2206	T2DM in primary care	NFS > 0.676 or FIB‐4 > 2.67	9	Advanced fibrosis Defined by VCTE LSM ≥ 8 kPa, MRE, liver biopsy ≥ F3, or definitive radiological evidence of hepatic cirrhosis	89	No	56.4% of the target group were referred to hepatology, 76% of the referred patients completed their hepatology visit	Poorly controlled T2DM was an independent risk factor for advanced fibrosis Sensitivity 56%, specificity 66%
Zhang et al. [25] Hong Kong & Malaysia	*n* = 533	T2DM and 18–70 years in primary care and outpatient clinic	FIB‐4 > 1.3 (< 65 year)/> 2.0 (≥ 65 year) or APRI > 0.5	4	Advanced fibrosis Defined by VCTE LSM ≥ 10 kPa, F3/F4 on histology, unequivocal radiological features of cirrhosis, or clinical, radiological or endoscopic evidence of portal hypertension	49	Yes (*n* = 528) Patients receiving standard care	33.3% of the high‐risk patients were referred to hepatology	Appropriate referral was increased from 3.1% in the control group to 33.3% in the intervention group
Ajmera et al. [19] USA	*n* = 403 (AGA pathway), *n* = 404 (AASLD guidance)	T2DM and 50–80 years in primary care and outpatient clinic	AGA pathway: FIB‐4 > 1.30 and VCTE‐LSM ≥ 8 kPa Or FIB‐4 > 2.67 AASLD guidance: FIB‐4 > 1.30 and ELF > 7.7 Or FIB‐4 > 2.67	AGA pathway: 6 AASLD guidance: 2	Advanced fibrosis Defined by MRE ≥ 3.63 kPa	AGA pathway: 11 AASLD guidance: 13	No	Not reported	False negative rates of 3.3% (AGA pathway) and 4.5% (AASLD guidance), 50% hepatology referral when using AASLD algorithm
Caussy et al. [26] France	*n* = 654	MASLD confirmed by steatosis on US, and 40–80 years, and T2DM or obesity in outpatient clinic	FIB‐4/VCTE LSM: FIB‐4 > 1.3 and VCTE‐LSM > 8 kPa FIB‐4/SWE LSM: FIB‐4 > 1.3 and SWE‐LSM ≥ 8 kPa FIB‐4/ELF: FIB‐4 > 1.3 and ELF > 9.8	FIB‐4/VCTE LSM: 7 FIB‐4/SWE LSM: 6 FIB‐4/ELF: 6	Advanced fibrosis Defined by hierarchical composite criterion of liver biopsy (≥ F3), MRE, or VCTE LSM ≥ 12.0 kPa	FIB‐4/VCTE LSM: 8 FIB‐4/SWE LSM: 10 FIB‐4/ELF: 12	No	N.A. given standardised research visit on the same day	False negative rates of 5% (FIB‐4/VCTE LSM), 6% (FIB‐4/SWE LSM), and 9% (FIB‐4/ELF)

Abbreviations: AASLD, American Association for the Study of Liver Diseases; AGA, American Gastroenterological Association; ALT, alanine transaminase; AST, aspartate transferase; BMI, body mass index; CRF, cardiometabolic risk factor(s); FIB‐4, fibrosis‐4 score; LSM, liver stiffness measurement; MASLD, metabolic dysfunction‐associated steatotic liver disease; MRE, magnetic resonance elastography; OR, odds ratio; SWE, shear wave elastography; T2DM, type 2 diabetes mellitus; UK, United Kingdom; US, ultrasound; USA, United States of America; VCTE, vibration‐controlled transient elastography.

^a^ Unless specified otherwise, advanced fibrosis refers to stage F3 or higher whereas significant fibrosis refers to stage F2 or higher.

### 3.3 Referral Rate

Seven pathways ultimately stratified patients into low‐ and high‐risk groups for advanced or significant fibrosis, while two added an intermediate‐risk category. Patients deemed low‐risk remained at the primary care setting/regular care, whereas patients at high‐risk were referred to the hepatologist. The approach to intermediate‐risk patients varied: in the study by Fox et al., these patients remained in primary care, whereas in the paper by Ajmera et al. they were referred to the hepatologist. In eight studies, the majority of patients (69.0%–98.0%) that entered the pathway were considered at low risk and remained under treatment with their treating physician (mostly general practitioner). The only study in which the majority of patients were referred to hepatology clinic was the AASLD care pathway in the study of Ajmera et al., with a referral rate of 50.5%. The NNS for referral to hepatology varied between 2 (Ajmera et al., following a two‐tier pathway using FIB‐4 and Enhanced Liver Fibrosis (ELF) in patients with T2DM in both primary and outpatient specialist care) to 19 (Mansour et al., a two‐tier pathway using FIB‐4 and VCTE‐LSM in patients with T2DM in primary care alone).

### 3.4 Fibrosis Detection

Eight studies reported the eventual number of patients with advanced disease, defined by their definition of either significant fibrosis (two studies) or advanced fibrosis (six studies). The definition of significant or advanced fibrosis varied widely between studies (see Table 1). The NNS to detect one case of significant fibrosis ranged from 12 to 40, to detect advanced fibrosis from 8 and 89. Additionally, two of the included studies calculated odds ratios (OR) for identification of advanced fibrosis by the implemented care pathway versus regular care [22, 24]. Mansour et al. found an OR of 6.7, and the pathway proposed by Srivastava et al. performed five times better (OR 4.9) as compared to standard care thereby achieving an 81% reduction in unnecessary referrals. The study by Ajmera et al., assessing a two‐tiered pathway in patients with T2DM using FIB‐4 and VCTE or ELF, demonstrated a low rate of misclassifying patients with advanced fibrosis as low risk (3.3% and 4.5% respectively). The same study found that the combination of FIB‐4 with ELF led to a higher hepatology referral rate compared to the combination of FIB‐4 and VCTE‐measurement, (50.4% vs. 18.1%); paradoxically, it also resulted in a higher false negative rate. The latter was also concluded on by Caussy et al., comparing false negative rates between algorithms using FIB‐4 and VCTE‐LSM (5%), FIB‐4 and SWE‐LSM (6%), and FIB‐4 and ELF (9%).

### 3.5 Attendance Rate

In the six studies reporting on attendance rate, dropout rates got increasingly higher as patients progressed further through the care pathways, with dropout rates being especially high at the step towards referral to the hepatologist. In cases where referral was recommended due to high risk for advanced disease, three studies reported that only about 50% of patients actually visited a hepatologist [20, 24, 27], while one study reported an even lower rate of 33.3% [25].

## 4 Discussion

In this study, we provide a state‐of‐the‐art review of the existing literature on clinical care pathways for MASLD. This review focused exclusively on MASLD and MASH, excluding other liver diseases such as alcoholic liver disease or viral hepatitis. As such, our approach is contrasted by that followed by Abeysekera and colleagues [28].

Nine studies conducted in seven countries were included, of which four evaluated a one‐tier pathway, and five evaluated a two‐tier pathway. Study sizes ranged from 162 to 2206 participants, encompassing a total of 8551 participants. FIB‐4 and VCTE were the most commonly used non‐invasive tests across all evaluated pathways. The NNS for referral to hepatology varied between 2 and 19. The NNS to detect significant fibrosis ranged from 12 to 40 and advanced fibrosis from 8 and 89, with inconsistencies in the definitions of fibrosis across the studies. The attendance rate declined as patients progressed through the pathways. Overall, all studies concluded that a one‐, or two‐tiered pathway for assessing fibrosis could effectively improve the identification of patients with advanced stages of MASLD and reduce both over‐ and under‐referrals to hepatologists. Based on the publications included in this review, this holds for both populations of patients with confirmed MASLD as well as those at risk based on metabolic comorbidities.

Until recently, screening for MASLD in high‐risk groups was not recommended and as a result most patients with advanced fibrotic MASLD were diagnosed following investigation of abnormal liver blood tests. In a survey of more than 100 primary care physicians, the vast majority (70%) indicated that they were unlikely to refer a patient to hepatology unless the patient had abnormal liver enzyme levels [29]. However, advanced fibrotic MASLD is often present even in patients with normal ALT levels [30, 31]. It can further be argued that, unless integrated into routine practice, following a clinical care pathway for MASLD may highly depend on local champions, for example, clinicians with a particular interest in the disease. We expect that with the publication of updated guidelines for MASLD incorporating screening methods and referral strategies in both primary, secondary, and tertiary care, awareness among care providers will increase. The advent of pharmacotherapy may tip the balance further in favour of screening in patients with cardiometabolic risk factors for MASLD [4].

The considerable variation in the NNS for referral to hepatology between the evaluated care pathways included in this review can—at least in part—be explained by the different design choices made. Screening in the general population will, by definition, result in higher NNS compared to screening in high‐risk populations (e.g., T2DM), due to the higher prevalence, age, or a priori probability of finding at‐risk patients in the latter group [32]. The lowest NNS for referral to hepatology—two—was found in the AASLD pathway from the study by Ajmera et al., where 50.5% of patients were referred. This is likely due to the fact that only patients aged 50–80 years were included in this pathway, while no age‐adjusted FIB‐4 cutoff was used. A known limitation of FIB‐4 is the inclusion of age in the calculation, resulting in the recommendation to use a higher cutoff for patients ≥ 65 years [33], although this approach is under debate [34]. These findings highlight the substantial influence of the target population, the choice of NITs, and their cut‐off values on the overall performance of the clinical pathway.

Ideally, NITs used in care pathways have high sensitivity and high negative predictive value. Yet, especially in primary care settings, both financial and geographical availability are equally import factors when choosing a suitable NIT for clinical use [35]. This may be an important reason for the abundant use of FIB‐4 compared to other NITs, in addition to the fact that FIB‐4 is now included in most guidelines [14, 36, 37]. The FIB‐4 score is readily accessible, as it relies on ALT, AST, and platelet counts—routine laboratory parameters that are easily obtainable in general practice and frequently assessed.

Nonetheless, some studies demonstrated that the diagnostic accuracy of FIB‐4 is limited, especially when T2DM is present [38, 39]. In the future, making more specialised NITs (such as VCTE‐measurement) readily available to primary care providers in a financially sustainable manner may lead to improved possibilities for screening at‐risk populations. In that light, taking into account regional variation in availability of diagnostic resources might prove to be a vital step in ensuring optimal care pathway performance.

In discussing NITs and their use in clinical care pathways for fibrotic MASLD, we should stress that alternative approaches and tests have been proposed beyond those predominantly used in the publications we included in this review. These include specialised biomarkers, for example, PROC3 and FibroTest on the one hand, and LSM‐based scores, for example, FAST and AGILE 3+ on the other hand [13]. Underrepresentation of such tests in the included studies might be explained by the notion that this review primarily aimed at including studies that prospectively assessed care pathways for MASLD, thereby mostly relying on previous designs that used more readily available and established NITs rather than the relative new tests mentioned above.

The NNS to detect one case of advanced disease, irrespective of the specific definitions employed in the articles under review, did also differ greatly between studies, ranging from 8 to 89. There was no consistent definition of advanced disease across the studies. Definitions varied considerably, both in terms of the fibrosis stage considered (significant versus advanced fibrosis) and the specific criteria used. For example, Fox et al. defined advanced fibrosis among others as a VCTE value ≥ 8 kPa, while Mansour et al. based their definition on F3 or F4 fibrosis on liver histology, radiological evidence, the presence of gastroesophageal varices, or overall clinical assessment. Moreover, most studies did not follow‐up on the patients who remained in primary care, making it impossible to determine how many were misclassified as having no risk of advanced fibrosis.

When assessing the performance of a care pathway, a clear trade‐off exists between maintaining high sensitivity on the one hand and minimising the number of false positives on the other. A commonly mentioned concern is that the implementation of a clinical care pathway could lead to a significant increase in referrals to secondary care, causing higher health care costs and reduced accessibility of care due to longer waiting lists. However, Srivastava et al. demonstrated that the use of their care pathway resulted in a 3% reduction in the proportion of cases diagnosed MASLD that were referred to secondary care per year, with only a modest increase in the total number of referrals. Moreover, they found this approach to be cost‐efficient with a reduced spend budget of 25.2% compared to standard care [40]. This finding is in line with multiple recent studies concluding on likely cost‐effectiveness of community‐based clinical care pathways for MASLD [41, 42, 43].

The review has several—sometimes overlapping—limitations. In general, the most important limitations can all be traced back to the critical notion that a universally accepted outcome framework for care pathways for MASLD is lacking. The absence of a clinical gold standard (e.g., liver biopsy or MRE) or widely accepted reference method for detecting advanced disease as well as the use of a reference method that is also incorporated in the pathway itself hampers the assessment of whether patients were rightfully referred. Therefore, it was not possible to determine sensitivity and specificity of all pathways. Although previous studies have shown that VCTE values ≤ 8 kPa and ≥ 12 kPa have high sensitivity and specificity for excluding and diagnosing advanced fibrosis respectively [44], LSM remains a surrogate for liver fibrosis and critical comments can be made about its use as reference method in several of the reviewed papers. Additionally, the absence of a control group in six out of nine included studies prevented us from comparing pathway performance to outcomes from regular care. These inconsistencies underscore the need for a universally accepted outcome framework for care pathways—ideally one that does not rely solely on liver biopsy.

Additionally, a high degree of heterogeneity exists between the study design of the included clinical care pathways, for example, the included patient population or the used NITs. Furthermore, several included studies had been conducted in different care settings, for example, primary care versus diabetology clinics. We aimed to compare pathways' clinical performance in terms of NNS for referral to hepatology and identification of either significant or advanced fibrosis. However, the fact that substantial differences existed in study context limited the degree of comparability.

Last, besides the care pathways evaluated by the papers included in this review, we are aware that several clinical care pathways exist in practice that have not been—or may never be—described in the published literature, thereby leaving a knowledge gap. However, by synthesizing and critically appraising the available evidence, this review still provides valuable insights into the range of evaluated pathways, identifies key areas for improvement, and may guide future standardisation and research.

Our findings underscore the necessity of evaluating comprehensive care pathways in larger and well‐defined population settings. Currently, several large‐scale European studies are underway to address this gap. The GRIP on MASH‐study, a large European care pathway implementation project, is enrolling 10 000 patients with at least one metabolic risk factor and incorporates the use of FIB‐4 and VCTE‐measurements. Another European study, the LiverScreen project, is screening 30 000 people in the general population to validate VCTE‐measurement as feasible screening strategy for liver fibrosis detection. Furthermore, the European LiverAim consortium intends to evaluate a screening program for fibrosis in 100 000 individuals from the general population, not only focusing on MASLD but taking into account other liver diseases as well, like alcohol‐related liver disease (ALD) and metabolic dysfunction and alcohol‐related steatotic liver disease (MetALD).

Future research on the development and implementation of clinical care pathways for MASLD should aim to address the general limitations mentioned above. Additionally, the increasing use of real‐world data will be instrumental in informing professional organisations and policymakers on the optimal organisation of MASLD care. In addition to large‐scale, real‐world studies on pathway performance, successful implementation also requires data on experience with and attitude towards implementation of care pathways, which should be gathered through survey‐based or qualitative research.

Data from studies conducted in substantially larger and more broadly defined cohorts than those reported to date are eagerly awaited, aligning with the key research agenda for MASLD proposed in the recent EASL‐EASD‐EASO clinical practice guideline. Investigating the distinct outcomes from screening high‐risk groups versus the general population also remains a crucial area of interest.

## 5 Conclusion

Clinical care pathways integrating NITs have the potential to optimise patient management and improve care coordination for MASLD, yet current evidence highlights significant heterogeneity in the design and application of MASLD care pathways. Present literature shows no consistent definition of advanced disease, underscoring the need for a universally accepted outcome framework for care pathways. Successful pathway implementation depends on achieving a balance between diagnostic accuracy, geographical accessibility, and financial feasibility.

Despite growing interest in risk stratification for fibrotic MASLD, large‐scale evidence supporting NIT‐based screening in high‐risk cardiometabolic populations remains limited. Addressing this critical research gap should be a priority, ensuring that care pathways are efficient, evidence‐based, and scalable for widespread clinical adoption.

## Author Contributions

Kirsi M.A. van Eekhout and Leonard D. Broekman were primarily responsible for the conceptualization, data curation, formal analysis, investigation, methodology, and drafting the original manuscript. Vivian D. de Jong, Maurice Michel, Rick Grobbee, Douglas Maya‐Miles, Manuel Romero‐Gómez, Jean Muris, Juan M. Mendive, Yasaman Vali, and Céline Fournier‐Poizat contributed to the writing through review and editing. Oscar H. Franco, Jörn M. Schattenberg, Manuel Castro Cabezas, and Maarten E. Tushuizen contributed to the conceptual development of the study and participated in reviewing and editing the manuscript. Adriaan G. Holleboom contributed to the conceptualization and manuscript revisions, and also provided overall supervision for the project.

## Funding

The GRIPonMASH project is supported by the Innovative Health Initiative Joint Undertaking (IHI JU) under grant agreement No 101132946. The JU receives support from the European Union's Horizon Europe research and innovation program and COCIR, EFPIA, EuropaBio, MedTech Europe, Vaccines Europe, and Mercodia Aktiebolag, Metadeq Limited, and Julius Clinical Research BV. Views and opinions expressed are, however, those of the author(s) only and do not necessarily reflect those of the aforementioned parties. Neither of the aforementioned parties can be held responsible for them.

## Conflicts of Interest

M.R. received a grant from Novonordisk, Siemens, Echosens and Teratechnologies, consulting fees from Ipsen and Boehringer Ingelheim, a payment for presentations or lectures from Novonordisk, Alphasigma, Roche and Gilead, and support for attending meetings from Gilead and Abbvie. J.M.M. received a payment for lectures of presentations from Novo Nordisk. J.M.S. served as consultant for Akero, Alentis, Alexion, Altimmune, Astra Zeneca, 89Bio, Bionorica, Boehringer Ingelheim, Boston Pharmaceuticals, Gilead Sciences, GSK, HistoIndex, Ipsen, Inventiva Pharma, Madrigal Pharmaceuticals, PRO.MED.CS Praha a.s., Kríya Therapeutics, Eli Lilly, MSD Sharp & Dohme GmbH, Novartis, Novo Nordisk, Pfizer, Roche, Sanofi, and Siemens Healthineers and has stock in Hepta Bio. CF is full time employee at Echosens. All other authors declare no conflicts of interest.

## Supporting information


**Supplementary S1.** Ovid MEDLINE search.

## Data Availability

No new data were generated in this study. Data sharing is not applicable to this article.
