# Supplementary material for: Care Pathways for Metabolic Dysfunction‐Associated Steatotic Liver Disease (MASLD): A State‐of‐The‐Art Review
Source: Liver Int. 2026 Mar 19;46(4):e70603. doi: 10.1111/liv.70603 (PMC13000678; doi:10.1111/liv.70603)
Supplement: Supplementary file 1 — Supplementary S1. Ovid MEDLINE search. [file LIV-46-e70603-s001.docx]

# Supplementary 1: Ovid MEDLINE search

| **Source:** | Ovid MEDLINE(R) ALL <1946 to January 19, 2026> |
| --- | --- |
|  |  |
| **#** | **Search** |
| 1 | Non-alcoholic Fatty Liver Disease/ |
| 2 | ((non-alcoholic adj3 (fatty liver disease or steatohepatitis)) or NAFLD or NASH).ab,kf,ti. |
| 3 | ((metabolic dysfunction-associated adj2 liver disease) or MASLD).ab,kf,ti. |
| 4 | (metabolic dysfunction-associated steatohepatitis or MASH).ab,kf,ti. |
| 5 | Liver Cirrhosis/ |
| 6 | liver cirrhos*.ab,kf,ti. |
| 7 | Fibrosis/ and Liver/ |
| 8 | liver fibros*.ab,kf,ti. |
| **9** | **or/1-8 [MASLD]** |
| 10 | exp Patients/ |
| 11 | (patient* or individual* or wom?n* or men or man or female* or person* or people* or group* or population* or client* or case* or recipient* or inpatient* or outpatient* or sufferer or resident* or participant* or adolescent*).ab,kf,ti. |
| **12** | **or/10-11 [Patients]** |
| 13 | Critical Pathways/ or exp Primary Health Care/ |
| 14 | ((care or critical or proactive or community or diagnostic* or refferal*) adj3 (path* or map* or flow* or track* or primary or guide* or screen* or detect*)).ab,kf,ti. |
| **15** | **or/13-14 [Care Pathways]** |
| **16** | **and/9,12,15 [MASLD & Patients & Care Pathways]** |
| 17 | exp Child/ or exp Infant/ or Pediatrics/ |
| 18 | (child* or infant* or kid or kids or underage or boy* or girl* or juvinile* or pediatric or baby or babies or newborn* or teen*).ab,kf,ti. |
| **19** | **or/17-18 [Children]** |
| **20** | **16 not 19 [Children Excluded]** |
| 21 | (exp animal/ not human/) or disease models, animal/ or exp animals, laboratory/ or exp rodentia/ or cattle/ or exp animals, genetically modified/ |
| 22 | ((animal adj3 (model* or experiment* or genetically modified)) or canine or dog or dogs or beagle* or feline or cat or cats or rodent* or rabbit* or mice or mouse or murine* or rat or rats).ab,kf,ti. |
| **23** | **or/21-22 [Animal filter]** |
| ***24*** | ***20 not 23 [Animals Excluded]*** |
| 25 | ("33613940" or "30965069" or "34847156" or "34179738" or "34139066" or "32414883" or "39280346").an. |
| 26 | 24 and 25 |
| 27 | 9 and 25 |
| 28 | 12 and 25 |
| 29 | 15 and 25 |
